# A proposed machine learning architecture for the subtyping, diagnosis, and characterization of Frontaltemporal Dementia (FTD)

**DOI:** 10.1002/alz70856_103689

**Published:** 2025-12-26

**Authors:** Ethan Wong, Liz Yuanxi Lee, Marcella Montagnese, Zoe Kourtzi, Timothy Rittman

**Affiliations:** ^1^ University of Cambridge, Cambridge, Cambridgeshire, United Kingdom; ^2^ University of Cambridge, Cambridge, United Kingdom; ^3^ Department of Clinical Neurosciences, University of Cambridge, Cambridge, Cambridgeshire, United Kingdom

## Abstract

**Background:**

Frontotemporal dementia (FTD) is characterized by a decline in cognitive capabilities including behavior, judgment and language. Classically, FTD is divided into three subtypes: behavioural variant (bvFTD), semantic variant (SD), and non‐fluent variant (nfvPPA). FTD and its subtypes often go misdiagnosed, emphasizing the need for robust computational tools that can more accurately diagnose/subtype the disease and characterize an individual's likely disease progression. Very little work has been done in developing clinically translatable machine learning (ML) tools to model FTD, with many currently available diagnostic and prognostic algorithms relying on non‐continuous metrics and clinical labels instead of capturing information in longitudinal patient trajectories.

**Method:**

We aimed to address these limitations by developing and validating a trajectory modeling approach that allows for enhanced characterization of an individual's cognitive progression. Specifically, we extended a Generalized Matrix Learning Vector Quantization (GMLVQ) machine learning algorithm previously successfully applied to Alzheimer's disease, optimizing and implementing it for FTD. We trained our models on the Neuroimaging in Frontotemporal Dementia (NIFD) dataset, comprising 288 FTD patients and 118 controls with baseline and longitudinal clinical, cognitive, and neuroimaging data (T1 weighted structural MRI). Utilizing Freesurfer, we extracted segmentation and parcellation data containing cortical thicknesses and volumes in all major cortical and subcortical regions yielding ∼180 neuroimaging features for modeling.

**Result:**

Our initial binary classification GMLVQ models for SD versus bvFTD/nfvPPA achieved a 94.4% accuracy. Areas like the left hemispheric (LH) temporal pole, the LH inferior, middle, and superior temporal gyri, and the LH fusiform gyrus appeared to be most influential in the model's decision. Extending our GMLVQ model towards the multi‐class classification of all three subtypes, we achieved a 79.9% accuracy, with most misclassifications relating to differentiating bvFTD and nfvPPA patients. Finally, to probe our GMLVQ model architecture's prospective performance amongst a broader neurodegenerative disease dataset, we fit a six‐class classifier for each FTD subtype alongside Alzheimer's disease, mild cognitive impairment, and control patient data (dervied from ADNI4), achieving an accuracy of 52.2%.

**Conclusion:**

Further model tuning for the multi‐class model is most needed; however, these preliminary results suggest that GMLVQ trajectory modeling shows promise for advancing the diagnosis and assessment of FTD.

